# Detection and Assay of Vitamin B-2 (Riboflavin) in Alkaline Borate Buffer with UV/Visible Spectrophotometry

**DOI:** 10.1155/2014/453085

**Published:** 2014-09-02

**Authors:** Ronald Bartzatt, Tasloach Wol

**Affiliations:** Durham Science Center, Chemistry Department, University of Nebraska, 6001 Dodge Street, Omaha, NE 68182, USA

## Abstract

The detection and assay of vitamin B-2 (riboflavin) was accomplished under aqueous conditions using sodium borate buffering at pH 7.52 conditions. The absorbance spectrum of riboflavin was determined at different pH values utilizing several buffers. The buffer at pH at 7.52 is followed by accurate and sensitive assay of riboflavin by spectrophotometer at 440 nm wavelength. Where indicated an origin solution (stock) was employed by dissolving sufficient vitamin to make a stock solution of 1.403 × 10^−4^ molar concentrations. Measurements of various aqueous solutions containing riboflavin were accomplished that included aqueous test samples, vitamin capsules/tablets, and water vitamin mixtures. A standard curve extended from 7.97 × 10^−7^ molar to 1.23 × 10^−4^ molar (a 154*x* folds spread in concentration). The equation of the line was *y* = 12545*x* (intercept at origin) with Pearson *r* correlation of 1.000 (*R* ^2^ = 1.000). Concentration of riboflavin assayed ranged from 3.00 × 10^−4^ gram per liter (0.30 ppm) to 0.0463 gram per liter (46.35 ppm). The B vitamin riboflavin can be assayed by UV/VIS spectrophotometer at 440 nm in aqueous media and using sodium borate buffer at pH 7.52. The assay can reach as low as 0.30 parts per million with high levels of accuracy and sensitivity.

## 1. Introduction 

Riboflavin (vitamin B-2) is an important B-vitamin that is found in a large variety of foods [1]. Vitamin B-2 is known to be the central component of the cofactor's flavin adenine dinucleotide (FAD) and flavin mononucleotide (FMN), which is necessary to all flavoproteins [1]. For this reason, vitamin B-2 is necessary for a large variety of cellular processes [1–3]. A deficiency of riboflavin has been shown to be associated with poor iron assimilation, an etiology link to anemia, and as a risk for cancer [1, 4]. As intake of riboflavin increases so does the urinary excretion clearance, with a steady maintained excretion increase as the intake continues, having an absorption half-life of 1.1 hours [1]. Riboflavin is an important participant in numerous diverse internal redox reactions as a part of metabolism and an inadequate intake of this vitamin would contribute to difficulties in intermediary metabolism [1]. A variety of clinical applications for riboflavin administration have been determined; these include (1) large dose intake to resolve infant neurological abnormalities, anemia, and visual impairment [5] and (2) prevention of migraines at high doses [6].

Riboflavin deficiency is found to be endemic in many regions of the world and that certain sections of populations in developed countries show a low intake with deficiency endemic in populations having diets lacking dairy products and meat [1]. If intake of riboflavin is poor during maternity status, then the infant is likely to be born riboflavin deficient [1].

Large-scale surveys conducted within the United States have reported noticeable riboflavin deficiencies among the elderly to be between 10% and 27% [1]. As to athletic performance, various studies have indicated that vigorous exercise can deplete riboflavin [1]. Riboflavin has been studied, with some positive results, to be an attractive candidate for reducing iron in heme proteins for the protection of tissues from oxidative injury [1].

Considering the substantial nutritional importance of riboflavin, the detrimental effects of deficiency, and potential for beneficial clinical use, then a broad diverse methodology to assay for the vitamin would be very efficacious. Taking advantage of the aqueous solubility of the vitamin, previous work has demonstrated that the vitamin can be estimated in mixtures that are also 0.1 normal in NaOH and with a double-beam ultraviolet-visible spectrometer [7]. Alternatively, a simultaneous determination of B-1, B-2, B-3, and B-6 was accomplished by reversed-phase C-18 column with high-performance liquid chromatography (HPLC) [8]. Fluorescence spectroscopy was successfully applied for vitamin B-2 determination having lesser sensitivity for detection [9]. Utilizing high-performance thin layer chromatography and various mobile phase solvents, such as 1-butanol-chloroform-acetic acid-ammonia-water, 7 : 4 : 5 : 1 : 1, or benzene-methanol-acetone-acetic acid, 70 : 20 : 5 : 5, or chloroform-ethanol-acetone-ammonia, 2 : 2 : 2 : 1, achieved some useful level of separation for riboflavin identification [10]. Other various liquid chromatography approaches were able to extract vitamins B-1 and B-2 from foods for determination [11, 12] and use reverse-phase chromatography with 254 nm detection [13], biosensor technology [14], ion-pair chromatography [15, 16], determination in dairy products [17], foodstuffs [18], detection of casein [19], total riboflavin in foods [20], riboflavin in infant formula [21, 22], and simultaneous thiamine, riboflavin, and niacin in food [23]. Methods of a riboflavin assay that utilize UV/visible spectrometry with solvation into aqueous media for speed, ease of application, low cost, high sensitivity, accuracy, and extensive application are very few.

The aim of this study is the presentation of a riboflavin assay methodology that has ease of application, high sensitivity, and accurate. Vitamin assay is an important and very large theater within areas of commercial production, food evaluation, and human-assembled merchandise manufacturing. The many types of uses that vitamins are applied to will require a continued investigation for determination of quantity and quality of vitamin presence. Furthermore, the potential benefits of the clinical applications of vitamin therapy require diverse and versatile methodologies for determining these and other vitamins.

## 2. Experimental 

### 2.1. Reagents and Instruments

Reagents utilized throughout this study were supplied by Sigma-Aldrich, P.O. Box  14508 St. Louis, MO 63178, USA. However, the vitamin riboflavin (FW = 376.37) was supplied by Eastman Chemical Company, 200 South Wilcox Drive, Kingsport, TN 37660, USA. For spectrophotometric analysis, a Spectronic 20D+ instrument was utilized with one-centimeter glass cuvettes.

### 2.2. Preparation of Standards and Test Samples

Riboflavin solid consists of orange crystals and produces a yellow aqueous solution. A stock solution of riboflavin was prepared by dissolving 0.0528 grams into one liter of distilled water making a concentration of 1.403 × 10^−4^ molar. This container was wrapped in aluminum foil to protect the riboflavin stock solution from light exposure. Stock solution of sodium borate buffer of 0.010 molar was prepared in distilled water at pH value of 7.52. A stock solution pH 7.36 utilizing phosphate-saline buffer (phosphate 6.6 millimolar and saline 0.154 molar) was prepared for evaluation of an absorbance spectrum as well as a stock solution of pH 9.01 utilizing Tris buffer (25 millimolar Tris base, 150 millimolar glycine) was made. The absorbance spectrum of vitamin B-2 was obtained at these pH values and buffers all with riboflavin at 1.403 × 10^−5^ molar. Absorbance values were obtained from 310 nm to 700 nm. Assay for vitamin B-2 (riboflavin) was ultimately accomplished at 440 nm wavelength which proved to be precise, reproducible, and accurate.

As many as ten mixtures were prepared for a standard curve and were made with desired aliquot volumes from the stock solution of riboflavin combined with sodium borate pH 7.52 buffer. Concentrations ranged from the highest at 1.23 × 10^−4^ molar to 7.97 × 10^−7^ molar with zero molar included within the linear equation. Many specimens were prepared using known amounts of riboflavin solubilized in pH 7.52 borate buffer and concentrations determined by standard curve for comparisons. To ascertain feasibility for measuring industrial or beverage aqueous vitamin mixtures, a set of pH 7.52 borate buffer examples was prepared at a wide range of riboflavin concentration. In the case of tablets or capsules, (1) the tablet was weighed, (2) then ground in mortar and pestle, (3) the dry amount of solid to be dissolved in volumetric flasks was weighed again (a separate aliquot of ground powder can be captured for other assays and the amount of the vitamin pill preparation that was dissolved for riboflavin assay was thus known), (4) the desired amount of solid is carefully placed in volumetric container and dissolved in distilled water, (5) further use requires the filtering out of insoluble solids through Whatman #1 filter paper, and (6) the filtered liquid is ready for assay or further dilution in the desired buffer. In all cases of test specimens to be assayed in this study, the material was dissolved in a known volume of pH 7.52 borate buffer (all assays were carried out at pH 7.52).

### 2.3. Software and Statistical Analysis

Computations performed for identification of numerical outlier's used Grubbs' test (extreme studentized deviate), means, and standard deviations were accomplished by Graphpad (http://www.graphpad.com/quickcalcs/). For statistical calculation of Pearson *r*, equation of a line from data, that is, graphed, and coefficient of determination (*R* ^*2*^) was taken from EXCEL (version 14.0.7106.5003, copyright 2010 Microsoft Corporation). Determination of statistical Pearson *r*, Mann-Whitney test, Kolmogorov-Smirnov test, Kruskal-Wallis test, and paired *t*-test was performed by PAST version 2.06 (copyright Hammer and Harper 1999–2011).

## 3. Results and Discussion

Riboflavin has a variable but low solubility in water, reaching approximately 1 milligram in 20 milliliters, with the variation due to differences in the internal crystalline structure of riboflavin [24]. The addition of NaCl can increase concentration in water as well as various solubilizing agents [1]. The solid consists of orange to orange-yellow crystals that are themselves not significantly affecting by light; however, when placed in aqueous solution (particularly alkaline conditions) the vitamin deteriorates rapidly [1]. For that reason, the stock solutions prepared for this study were kept stored at room temperature and with aluminum foil wrapping of the container to protect from light.

The riboflavin solid is easily handled and can be furthered ground to fine powder by mortar and pestle, a useful action for dissolving in water if the solid consists of large chunks. The solid is sufficiently soluble in distilled water to reach a concentration for the stock of 0.0001403 molar (0.0528 grams in one liter or 5.28E-05 grams in one milliliter).

**Figure 1:**
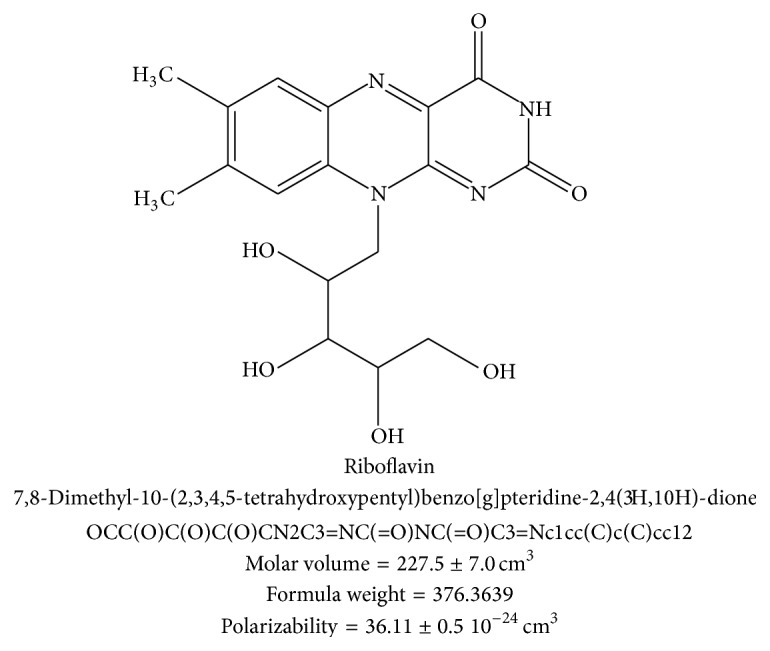
Molecular structure of vitamin B-2 with formula nomenclature, SMILES notation, molar volume, formula weight, and polarizability.

The molecular structure of vitamin B-2 consists of a flavin ring component that is also responsible for the yellowish color [1]. The molecular structure also contains four hydroxyl groups (–OH) covalently bond to an aliphatic chain that is in turn attached to the flavin ring moiety, shown in Figure 1. Several molecular properties are shown: molar volume (227.5 cm^3^), formula weight (376.36), polarizability, IUPAC scientific, and SMILES notation. Note that other members of the vitamin B group vitamins have significantly different molecular structures.

**Figure 2:**
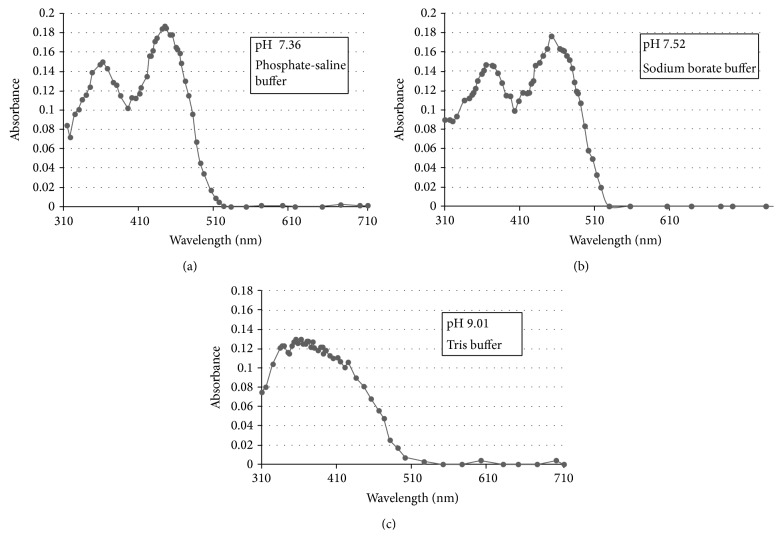
Absorbance spectrum of riboflavin at various pH and buffer. (a) is pH 7.36 utilizing phosphate-saline buffer (phosphate 6.6 millimolar and saline 0.154 molar) with riboflavin at 1.403 × 10^−5^ molar. (b) is pH 7.52 utilizing sodium borate buffer at 0.010 molar and riboflavin at 1.403 × 10^−5^ molar. (c) is pH 9.01 utilizing Tris buffer (25 millimolar Tris base and 150 millimolar glycine) with riboflavin at 1.403 × 10^−5^ molar.

To reach a conclusion on the appropriate buffering of aqueous solutions to be assayed a series of the absorbance spectra was obtained utilizing different buffering reagents at different values of pH in solution. These included a phosphate-saline system at pH 7.36, sodium borate at pH 7.52, and Tris-glycine buffering system at pH 9.01. These are presented in Figure 2 for comparison.

All absorbance spectra were accomplished in similar cuvettes and spectrometer. All absorbance spectra were obtained with riboflavin at a concentration of 1.403 × 10^−5^ molar. The absorbance spectrum spanned the wavelength range from 310 nm to 700 nm. The absorbance peak at 440 nm is of interest for quantitation of the vitamin in this study. At 440 nm wavelength the phosphate-saline buffer (pH 7.36) provided a molar extinction coefficient (molar absorption coefficient and molar absorptivity) of *ε* = 13114.8 liter/mole. The borate buffer system (pH 7.52) provided a molar extinction coefficient of *ε* = 12544.55 liter/mole. The Tris-glycine buffer system (pH 9.01) resulted in a molar extinction coefficient of *ε* = 6486.1 liter/mole, which was a much lower numerical value than that for the other buffering systems, and the spectrum obtained did not resolve a distinct peak at the desired wavelength of 440 nm. The subsequent assay was accomplished in the borate buffering system and at pH 7.52. Distinct absorbance peaks were identified for the phosphate-saline and borate buffering systems. For the phosphate-saline buffering, another absorbance peak was identified at 362 nm and for the borate buffering, a second large peak was identified at 360 nm.

The buffering system chosen was the borate pH stabilization at pH 7.52, which held pH very well and easy to handle. The formation of standard curves was easily accomplished at 440 nm from a range of 7.97 × 10^−7^ molar (3.00 × 10^−4^ grams per liter or 0.3 parts per million or 3.00 × 10^−7^ grams per milliliter) to 1.23 × 10^−4^ molar (0.0463 grams per liter or 46.4 parts per million or 4.63 × 10^−5^ grams per milliliter). This is extremely high sensitivity (to tenths of parts per million) under aqueous conditions and buffering by a common buffering system of sodium borate.

This buffering system provided very stable readings of test samples and others. The standard curve encompasses a 154*x* range in molar values. The equation of the line was *y* = 12545*x* (including origin) and with a correlation coefficient of essential *r* = 1.000 (a very strong positive relationship), with a coefficient of determination* R* ^*2*^ = 1.000. The standard deviation of slope is 0.66 and the 95% confidence interval for the slope is from 12543 to 12545.

Test samples and preparations of tablets or capsules containing riboflavin were prepared in aqueous solution only and having the sodium borate buffer, which stabilized pH strongly and there was no problems encounter. The buffer concentration ranged in concentrations from 1.00 × 10^−3^ molar to 1.50 × 10^−3^ molar, which is a very versatile attribute of using sodium borate as the buffering system. When using the single-beam UV/VIS spectrometer 20D+ the zero absorbance adjustment required only a plain water sample having the borate buffer at the same concentration as test samples. This method worked very well and with no difficulties. Therefore, buffer concentration can be adjusted according to solution parameters and objectives.

**Figure 3:**
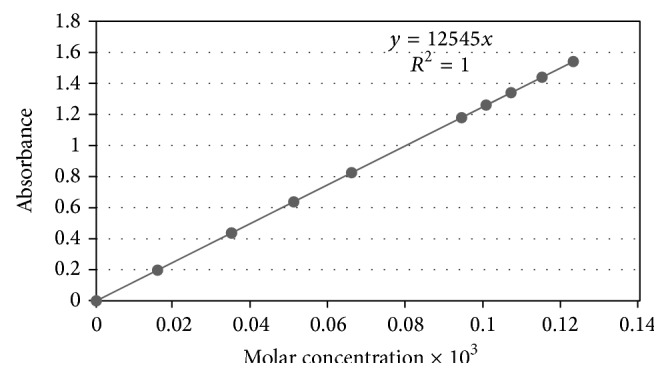
Standard curve. To determine concentration of test samples and commercial tablets/capsules the standard curve was formed and ranged from 7.97 × 10^−7^ molar to 1.23 × 10^−4^ molar. The concentrations are a range of 154*x* in molar values, an extremely broad range in linearity and expanse of concentration. The equation of line became *y* = 12545*x* with correlation coefficient of essential *r* = 1.000 and with coefficient of determination* R* ^*2*^ = 1.000. The standard deviation of slope is 0.66 and 95% confidence interval of slope is from 12543 to 12545.

The sample absorbance values were highly stable and reproducible. Having established the solution parameters to demonstrate reproducibility of preparation and buffering system, Table 1 shows repeat samples at two different concentration levels (low and high) comparing the molarity determined from a stock solution to the concentration obtained from the generation of a standard curve (see Figure 3).

Viewing Table 1 for test samples from 1 to 12 at low concentration of 3.488 × 10^−5^ molar (by stock solution determination) is compared to values obtained from the standard curves. Note that percent recovery is essentially thorough with an average of 100% (standard deviation = 0.95%, median = 100%, maximum = 101%, minimum = 98.3%, and skewness = −0.67 indicating moderate skewness to the left) [25]. The comparison of molarities of the samples from 1 to 12 shows by Mann-Whitney test to have equal medians (*P* = 0.47) and by *t*-test to have equal means (*P* = 0.93), and the two sets of molarities have equal medians by Kruskal-Wallis test (*P* = 0.49). Likewise, for the high concentration repeat test samples from 13 to 24 at 0.00008513 molar, the values obtained by standard curve for comparison to stock solution show a very high percent recovery average of 99.9% recovery (standard deviation = 1.2, maximum = 101%, minimum = 97%, and skewness = −1.3 indicating skewness to the left) [25]. For test samples from 13 to 24, the *t*-test shows equal means (*P* = 1.0); Mann-Whitney test shows equal medians (*P* = 0.43), equal medians by Kruskal-Wallis test (*P* = 0.49), and equal medians by Wilcoxon test (*P* = 0.52). Essentially the reproducibility is extremely high by standard curve and statistical analysis [25].

Another evaluation of test samples in aqueous solution with the borate buffer system also resulted in highly accurate and reproducible values of molarity. Presented in Table 2 are examples having a very broad range in apparent vitamin B-2 content, assayed by spectrometer and standard curve and compared to molarity based on a stock solution origin.

Comparison of calculated molarity from origin of stock solution to concentration obtained from a standard curve shows again to be very high in similarity and accuracy. For molarity values derived from the standard curve generated the range of molarity values vary from the minimum of 1.275 × 10^−6^ molar to the maximum of 1.147 × 10^−4^ molar (a 90*x* range in molarity numerical values). Compared to origin of stock solution molarity the values are highly accurate and reproduced. By the comparison of origin of molarity and standard curve molarity values, the mean of the two sets is the same by 2-sample *t*-test (*P* = 0.99), and the two sets have equal medians by Mann-Whitney test (*P* = 0.89). Furthermore, by the Kurskal-Wallis test the two sets have equal medians (*P* = 0.89). Both sets of molarity data have the same distribution by Kolmogorov-Smirnov test (*P* = 1.0).

The methodology outlined in EXPERIMENTAL shows an approach for preparation and assay of vitamin B-2 content from commercial tablets and capsules. Handling is done appropriately for material determined for human consumption, (i.e., storage at 4°C and handling by forceps to avoid grease and debris accumulation on the sample). Once the tablet or capsule is solubilized then the mixture is studied within a 24-hour period or stored at 4°C until used (however, samples stored should be examined for contaminating microbe growth).

For tablet analysis, it becomes necessary to grind the sample in a clean mortar and pestle before solubilizing into distilled water (the borate buffering can be added when aliquots are taken for actual analysis). Hitherto then, the commercial tablet showed a content of 8.80 mg total of vitamin B-2 with the industrial label claiming 7.5 mg total.

However, in the case of capsules, the material may already be in the form of particulate powder but can have small chunks requiring grinding in mortar and pestle (which enhances solubilizing). Likewise, for capsule sample from commercial entity the derived amount of vitamin B-2 came to 72 mg total of B-2 with the manufacturer claim at 100 mg. Therefore, the method can be applied to commercial products to evaluate for validation purposes and/or observation for adulteration of product.

Similarly, the B vitamins are placed into aqueous solution for in-house quality control, validation, and commercial sale to the public. Concentrations of riboflavin can be examined at these levels and usage. For presentation, a representative set of vitamin B-2 waters is obtained and test for content conducted as previously described.

Looking at Table 3, the values of molarity vary in a broad range from a minimum of 2.232 × 10^−6^ molar to a maximum of 3.555 × 10^−5^ molar, which is a 16*x* folds range in numerical values of molarity. Looking at the percent recovery it is starkly clear that the percent recovery is extremely high and indicating excellent accuracy. The mean value of percent recovery is 99.9% with a standard deviation of only 0.051. The minimum percent recovery is 99.9% with a maximum of 100%.

When comparing molarity from origin to molarity numerical values derived from standard curve generation the match of values is essentially agreed. By Mann-Whitney test, the two sets have equal medians (*P* = 0.86) and by Kruskal-Wallis test the two sets have the same medians (*P* = 0.86). Furthermore, by the Kolmogorov-Smirnov test, the two populations are taken from sets having equal distribution numerically (*P* = 1.0) [25]. The two populations have the same means by the *t*-test (*P* = 0.99). Consequently it is easy to conclude that the assay is representative and accurate.

The commercial usage of B vitamins will increase and applications of assay targeting these nutrients will be necessary. This methodology approach is simple and applicable for verification, quality control for industrial usage, surveillance for product adulteration, and verification purposes in distribution as medicaments. New approaches of methodology will benefit the versatile and successful creation of new commercial products as well as clinical application for treatment of many types of ailments.

Presented here is a versatile yet simple approach methodology that is accurate and reproducible. The method makes use of common spectrometer instrumentation and well-known buffering systems. In addition, the technique is making use of proven physicochemical character of the vitamin, such as solubility and stability. The investigation of novel techniques of an assay will be beneficial commercially and scientifically.

## 4. Conclusion

The measurements of various types of aqueous mixtures that contain riboflavin were accomplished; these include aqueous test samples, vitamin capsules/tablets, and water vitamin mixtures. This approach is fast, broadly applicable due to the aqueous solubility of riboflavin, easily understood, easily applied, facile for use in quality control, and efficient for process monitoring for commercial manufacturing. A standard curve extended from 7.97E-07 molar to 1.23E-04 molar (a 154*x* spread in concentration). Concentration of riboflavin assayed ranged from 0.000300 gram per liter (0.30 parts per million) to 0.0463 gram per liter (46.3 parts per million). Therefore, the B-2 vitamin (riboflavin) can be assayed by UV/VIS spectrophotometer at 440 nm in aqueous media and using borate buffer at pH 7.52. Sets of molarity data were shown to have equal means, medians, and population distribution by the appropriate statistic tests. Riboflavin determinations utilizing these conditions show high levels of accuracy and sensitivity.

## Figures and Tables

**Table 1:** Percent recovery vitamin B-2 for repeated samples for evaluation.

Test sample	Molarity	Molarity by standard curve	Percent recovery
1	3.49 × 10^−5^	3.46 × 10^−5^	99.2
2	3.49 × 10^−5^	3.48 × 10^−5^	99.7
3	3.49 × 10^−5^	3.48 × 10^−5^	99.7
4	3.49 × 10^−5^	3.43 × 10^−5^	98.3
5	3.49 × 10^−5^	3.51 × 10^−5^	101
6	3.49 × 10^−5^	3.46 × 10^−5^	99.2
7	3.49 × 10^−5^	3.51 × 10^−5^	101
8	3.49 × 10^−5^	3.51 × 10^−5^	101
9	3.49 × 10^−5^	3.49 × 10^−5^	100
10	3.49 × 10^−5^	3.54 × 10^−5^	101
11	3.49 × 10^−5^	3.51 × 10^−5^	101
12	3.49 × 10^−5^	3.51 × 10^−5^	101
13	8.51 × 10^−5^	8.45 × 10^−5^	99.3
14	8.51 × 10^−5^	8.61 × 10^−5^	101
15	8.51 × 10^−5^	8.53 × 10^−5^	100
16	8.51 × 10^−5^	8.25 × 10^−5^	97
17	8.51 × 10^−5^	8.61 × 10^−5^	101
18	8.51 × 10^−5^	8.45 × 10^−5^	99.3
19	8.51 × 10^−5^	8.45 × 10^−5^	99.3
20	8.51 × 10^−5^	8.61 × 10^−5^	101
21	8.51 × 10^−5^	8.61 × 10^−5^	101
22	8.51 × 10^−5^	8.53 × 10^−5^	100
23	8.51 × 10^−5^	8.61 × 10^−5^	101
24	8.51 × 10^−5^	8.45 × 10^−5^	99.3

**Table 2:** Percent recovery for samples based on vitamin B-2 content.

Test sample	Origin molarity	Molarity by standard curve	Percent recovery
1	1.28 × 10^−6^	1.28 × 10^−6^	100
2	2.63 × 10^−6^	2.63 × 10^−6^	99.9
3	4.94 × 10^−6^	4.94 × 10^−6^	99.9
4	6.86 × 10^−6^	6.86 × 10^−6^	99.9
5	8.53 × 10^−6^	8.53 × 10^−6^	99.9
6	1.03 × 10^−5^	1.03 × 10^−5^	100
7	1.23 × 10^−5^	1.23 × 10^−5^	99.9
8	1.40 × 10^−5^	1.40 × 10^−5^	99.9
9	1.58 × 10^−5^	1.58 × 10^−5^	100
10	1.71 × 10^−5^	1.71 × 10^−5^	99.9
11	1.95 × 10^−5^	1.95 × 10^−5^	99.9
12	2.07 × 10^−5^	2.07 × 10^−5^	99.9
13	2.23 × 10^−5^	2.23 × 10^−5^	99.9
14	2.37 × 10^−5^	2.37 × 10^−5^	99.9
15	9.25 × 10^−5^	9.25 × 10^−5^	99.9
16	8.85 × 10^−5^	8.85 × 10^−5^	100
17	8.61 × 10^−5^	8.61 × 10^−5^	100
18	8.33 × 10^−5^	8.33 × 10^−5^	100
19	8.05 × 10^−5^	8.05 × 10^−5^	100
20	7.73 × 10^−5^	7.73 × 10^−5^	100
21	7.37 × 10^−5^	7.38 × 10^−5^	100
22	7.06 × 10^−5^	7.05 × 10^−5^	99.9
23	6.78 × 10^−5^	6.78 × 10^−5^	99.9
24	6.43 × 10^−5^	6.42 × 10^−5^	99.9
25	1.12 × 10^−4^	1.12 × 10^−4^	99.9
26	1.15 × 10^−4^	1.15 × 10^−4^	99.9
27	6.43 × 10^−5^	6.42 × 10^−5^	99.9
28	6.58 × 10^−5^	6.58 × 10^−5^	99.9
29	6.66 × 10^−5^	6.66 × 10^−5^	100
30	6.98 × 10^−5^	6.98 × 10^−5^	99.9
31	7.29 × 10^−5^	7.29 × 10^−5^	100

**Table 3:** Percent recovery for aqueous vitamin waters for comparison.

Test sample	By origin molar	Molar by standard curve	Percent recovery
1	2.23 × 10^−6^	2.23 × 10^−6^	100
2	2.79 × 10^−6^	2.79 × 10^−6^	100
3	3.03 × 10^−6^	3.03 × 10^−6^	100
4	3.19 × 10^−6^	3.19 × 10^−6^	100
5	3.59 × 10^−6^	3.59 × 10^−6^	100
6	4.31 × 10^−6^	4.31 × 10^−6^	100
7	5.90 × 10^−6^	5.90 × 10^−6^	100
8	6.46 × 10^−6^	6.46 × 10^−6^	100
9	7.89 × 10^−6^	7.89 × 10^−6^	99.9
10	9.81 × 10^−6^	9.80 × 10^−6^	99.9
11	1.21 × 10^−5^	1.20 × 10^−5^	99.9
12	1.35 × 10^−5^	1.35 × 10^−5^	100
13	1.35 × 10^−5^	1.35 × 10^−5^	100
14	1.53 × 10^−5^	1.53 × 10^−5^	99.9
15	1.78 × 10^−5^	1.78 × 10^−5^	99.9
16	1.96 × 10^−5^	1.96 × 10^−5^	99.9
17	2.02 × 10^−5^	2.02 × 10^−5^	99.9
18	2.37 × 10^−5^	2.37 × 10^−5^	99.9
19	2.78 × 10^−5^	2.78 × 10^−5^	99.9
20	3.16 × 10^−5^	3.16 × 10^−5^	99.9
21	3.56 × 10^−5^	3.56 × 10^−5^	99.9

## Abbreviations

UV/VIS: Ultraviolet visible
SMILES: Simplified molecular-input line-entry system
FAD: Flavin adenine dinucleotide
FMN: Flavin mononucleotide
HPLC: High-performance liquid chromatography
IUPAC: International Union of Pure and Applied Chemistry
nm: Nanometers.
